# Enhancing the throughput and multiplexing capabilities of next generation sequencing for efficient implementation of pooled shRNA and CRISPR screens

**DOI:** 10.1038/s41598-017-01170-z

**Published:** 2017-04-21

**Authors:** Md. Fahmid Islam, Atsushi Watanabe, Lai Wong, Conor Lazarou, Frederick S. Vizeacoumar, Omar Abuhussein, Wayne Hill, Maruti Uppalapati, C. Ronald Geyer, Franco J. Vizeacoumar

**Affiliations:** 1 Department of Biochemistry, University of Saskatchewan, Saskatoon, S7N 5E5 Canada; 2 Department of Pathology, University of Saskatchewan, Saskatoon, S7N 0W8 Canada; 3 Department of Hematology, Nephrology and Rheumatology, Graduate School of Medicine, Akita University, Akita, Japan; 4 College of Pharmacy and Nutrition, University of Saskatchewan, Saskatoon, S7N 5C9 Canada; 5 Cancer Research, Saskatchewan Cancer Agency, 107 Wiggins Road, Saskatoon, S7N 5E5 Canada

## Abstract

Next generation sequencing is becoming the method of choice for functional genomic studies that use pooled shRNA or CRISPR libraries. A key challenge in sequencing these mixed-oligo libraries is that they are highly susceptible to hairpin and/or heteroduplex formation. This results in polyclonal, low quality, and incomplete reads and reduces sequencing throughput. Unfortunately, this challenge is significantly magnified in low-to-medium throughput bench-top sequencers as failed reads significantly perturb the maximization of sequence coverage and multiplexing capabilities. Here, we report a methodology that can be adapted to maximize the coverage on a bench-top, Ion PGM System for smaller shRNA libraries with high efficiency. This ligation-based, half-shRNA sequencing strategy minimizes failed sequences and is also equally amenable to high-throughput sequencers for increased multiplexing. Towards this, we also demonstrate that our strategy to reduce heteroduplex formation improves multiplexing capabilities of pooled CRISPR screens using Illumina NextSeq 500. Overall, our method will facilitate sequencing of pooled shRNA or CRISPR libraries from genomic DNA and maximize sequence coverage.

## Introduction

Recent advancements in sequencing technologies and their applications in functional genomics have significantly broadened our understanding of cellular functions and our ability to perform translational science. These technologies often involve the sequencing of a pool of ‘molecular barcodes’ that are unique in nature. For example, large-scale, genome-wide screens using pooled shRNA or CRISPR libraries query the genome and subsequent sequencing identifies the unique shRNA or sgRNA sequences that affect cell viability^1–6^. Such methods are increasingly applied to identify therapeutically relevant synthetic lethal targets^4–11^ or cancer-specific essential genes^2, 3, 12–20^. These novel interactions reveal potential targetable vulnerabilities of malignant cells and have resulted in the initiation of several clinical trials in the recent past (NCT01791309; NCT01750918; NCT01719380). Similarly, next generation sequencing technologies are also used in combinatorial techniques such as phage display, mRNA display, yeast display, and aptamer libraries^21–25^. A common theme in all of these sequencing reactions is that they depend on mixed-oligo PCR reactions wherein unique reads are binned by molecular barcodes distinctively associated with each sequence, allowing multiplexing.

While these sequencing methods are increasingly used in large core facilities, there are a number of challenges that impede their widespread usage in standard labs where cost-effective bench-top sequencers could be routinely employed. One reason for this is that most of these libraries are extremely large and these instruments do not provide enough usable reads required for sequence coverage. The availability of sub-libraries in certain assays that target a small subset of genes (such as ion channels, the kinome, etc.) can alleviate this issue and enhance the feasibility of using low-to-medium throughput sequencers. However, the formation of secondary structures and mixed heteroduplex template results in a major challenge as these structures reduce the number of useable sequences in a technology, which already experiences limited throughput^26^. The development of methods to mitigate sequencing failures will not only enhance the routine application of these techniques in standard labs, but will also increase the throughput and multiplexing capabilities in large core facilities.

**Figure 1:**
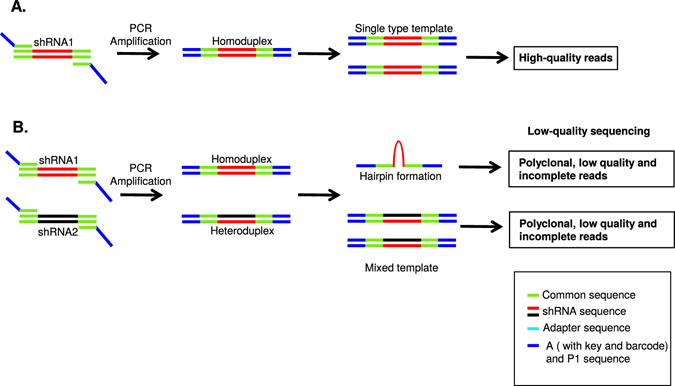
Schematic depicting challenges with shRNA library sequencing. (**A**) Schematic showing expected PCR product when amplifying a mixed-oligo library. (**B**) Schematic showing the formation of secondary structures (hairpin structure) and heteroduplex formation (mixed template due to primer shortage during high number PCR cycles), resulting in low quality sequencing reads.

Sequencing failure primarily occurs due to the formation of two structures: heteroduplex and hairpin (Fig. 1). The formation of heteroduplex is common when sequencing a library of DNA variants derived from the same parent or closely related templates. Particularly during PCR amplification of mixed-oligos, annealing of similar types of library sequences results in heteroduplex formation when there is a primer shortage^26–33^. This heteroduplex usually contaminates the intended library and reduces the quality of sequencing due to incomplete, low quality, and polyclonal reads. Hairpin structures result from palindromic sequences, and can also lead to incomplete, low quality, and polyclonal reads^26, 34–37^.

Here, we describe a method that successfully overcomes next generation sequencing issues related to hairpin and/or heteroduplex formation and maximizes library coverage. To prevent shRNA hairpins, we removed half of the hairpin by digesting the loop region with a restriction enzyme and ligating a small adapter; this was found to significantly reduce sequencing failure. We also show that reduced PCR cycles and the usage of magnetic beads for purification maximized our throughput and substantially diminished the number failed sequences through the reduction of heteroduplex formation. We optimized our methods using a minimally pooled shRNA library consisting of ~15,000 unique shRNA clones. We also performed additional validation assays of our method by applying to a pooled CRISPR library^19^.

## Methods

### Lentivirus preparation and viral transduction

Transfections were carried out using X-tremeGENE 9 (Roche) as per the manufacturer’s instructions. Pooled lentivirus expressing shRNAs were generated by transfecting HEK293T cells with psPAX2, pMD2.G, and pLKO.1 with library plasmids. Media was replaced 18 hours after transfection with DMEM containing 20% (w/v) bovine serum albumin (BSA) and lentivirus was collected after 24 and 48 hours. Transducing cells with pooled lentivirus caused stable knockdowns. Transduced cells were selected with 2 µg/ml Puromycin (Thermo Scientific) for 48 hours and passaged for subsequent generations as previously described^5, 6, 38^.

### Amplification of shRNA library from genomic DNA

**Figure 2:**
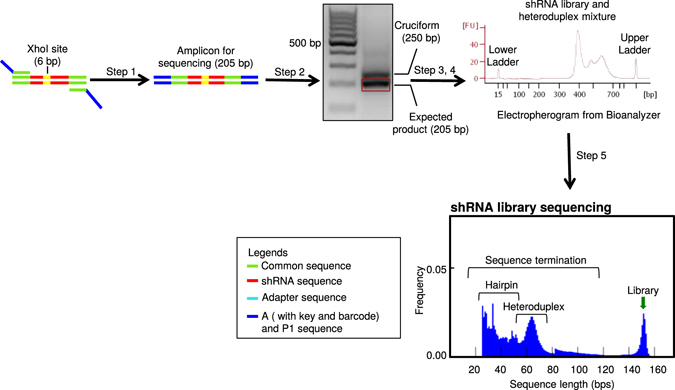
Steps involved in shRNA library sequencing. Workflow showing steps involved in sequencing shRNA library from the genomic DNA. Step1: PCR amplification of shRNA library from gDNA. Step 2: PCR purification of the amplified library. Step 3: Gel extraction of the 205 bp library amplicon. Step 4: Quality assessment of the library using a Bioanalyzer. Step 5: shRNA library sequencing using Ion Torrent platform. Representative agarose gel, electropherogram from the Bioanalyzer and read-length histogram from Ion Torrent are shown. The sequence length is plotted in the X-axis and the frequency is plotted in the Y-axis. The shRNA library is 152 bp, excluding the A and P1 sequence.

**Figure 3:**
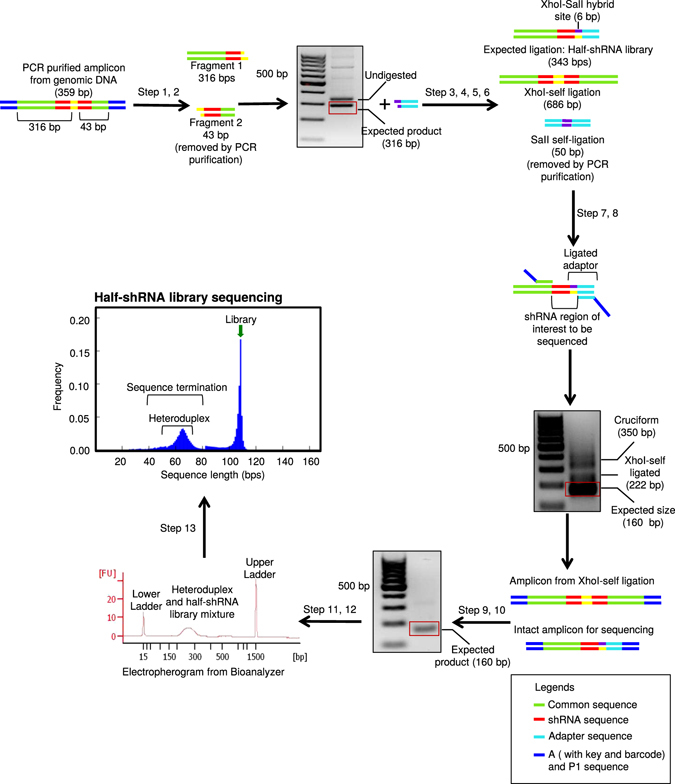
Elimination of the hairpin in shRNA library. Workflow showing steps to eliminate hairpin in the shRNA library. Step 1: *XhoI* digestion of purified PCR amplicon from gDNA. Step 2: PCR purification of *XhoI-*digested product. Step 3: Gel extraction of expected 316 bp *XhoI-*digested amplicon. Step 4: Preparation of the *SalI* adapter. Step 5: Adapter ligation to *XhoI-*digested amplicon. Step 6: PCR amplification of the ligated product. Step 7: PCR barcode labeling of the ligated product. Step 8: PCR amplification of the barcoded PCR product. Step 9: *XhoI* digestion of the amplicon from *XhoI* self-ligated product. Step 10: PCR amplification after digestion of amplicon from *XhoI* self-ligation. Step 11: Gel extraction of the 160 bp barcoded product. Step 12: Quality assessment of the barcoded product on the Bioanalyzer. Step 13: Half-shRNA library sequencing on Ion Torrent platform. Representative agarose gel, electropherogram from the Bioanalyzer and read-length histogram from Ion Torrent are shown. The sequence length is plotted in the X-axis and the frequency is plotted in the Y-axis. The half-shRNA library is 108 bp excluding A and P1 sequence.

Genomic DNA was isolated from cells using the QIAamp DNA Blood Maxi Kit (QIAGEN) according to manufacturer’s protocol. DNA concentration and purity were quantified using NanoDrop™ 2000/2000c (Eppendorf) and stored at −20 °C. For shRNA sequencing, Ion Torrent primers were directly used to amplify the library using oligonucleotides 1 and 2 (Table 1) from genomic DNA (Fig. 2). For half-shRNA sequencing, the shRNA library was amplified with a different primer set such that a larger amplicon of 359 bp was generated using oligonucleotides 3 and 4 (Table 1). When digested with *XhoI*, the larger 316 bp digestion product was large enough that it was easily separated from the smaller 43 bp digestion product, which was removed during PCR clean up (Fig. 3). To perform the PCR amplification with both set of primers, 160 µl of 10X Pfx Amplification Buffer (Invitrogen), 160 µl of 10X PCR_X_ Enhancer Solution (Invitrogen), 24 µl of 10 mM dNTPs (Thermo Scientific), 30 µl of 25 µM Forward Primer, 30 µl of 25 µM Reverse Primer, 24 µl of 50 mM MgSO_4_ (Invitrogen) and 12 µl of Platinum™ Pfx DNA Polymerase (Invitrogen) were added together with 20 µg of genomic DNA template. DNAase-free ddH_2_O was added to make the final volume to 800 µl. The entire 800 µl was divided into 16 reactions with each reaction as a 50 µl aliquot and PCR was done using a Thermal Cycler (Applied Biosystems). The temperature profile for the PCR was set as, 3 min at 98 °C, 30 cycles of amplification (10 sec at 98 °C, 15 sec at 55 °C, 15 sec at 72 °C) and 5 min at 72 °C. We used 20 µg template to ensure enough representation of each shRNA from the library during the amplification. PCR reactions were pooled together and purified using the GeneJET PCR Purification Kit (Thermo Scientific) according to manufacturer’s protocol and eluted in 100 µl DNAase-free ddH_2_O. DNA concentration and purity were quantified using NanoDrop™ 2000/2000c. A small sample (2 µl) of the PCR product (with 6X DNA Gel Loading Dye, Thermo Scientific) was run at 90 V for 45 minutes in a Gel Electrophoresis Apparatus (Bio-Rad) using a 2% UltraPure™ Agarose gel (Thermo Scientific) stained with SYBR® Safe DNA Gel Stain (Thermo Scientific) in 1X TAE (Thermo Scientific). The 205 bp (shRNA sequencing) and 359 bp (half-shRNA sequencing) expected bands were detected on Gel Doc™ XR +Imager (Bio-Rad) using 100 bp DNA Ladder (Thermo Scientific) (Figs 2 and 3). About, 2 µg of the 205 bp product was resolved (with 6X DNA Gel Loading Dye) at 90 V for 45 minutes in a Gel Electrophoresis Apparatus using 2% low-melting UltraPure™ Agarose (Thermo Scientific) and the gel was stained with SYBR® Safe DNA Gel Stain in 1X TAE. The 205 bp expected band was excised on a UV Transilluminator (Fisher Scientific) (Fig. 2). The expected product was purified by QIAquick Gel Extraction Kit (QIAGEN) according to manufacturer’s protocol and eluted in 25 µl DNAase-free ddH2O. DNA concentration and purity were quantified using NanoDrop™. The 359 bp product was used in the next step for making half-shRNA. The optimization of PCR cycle number for amplification of shRNA library from genomic DNA with Ion Torrent primers is shown in Supplementary Fig. S1A.

**Table 1:** Oligonucleotides for shRNA library preparation (Integrated DNA Tech.).

ID	Oligonucleotide (5′-3′)	Sequences
1.	Ion Torrent F.P. (A primer)	*CCATCTCATCCCTGCGTGTCTCCGAC* ^*1*^ TCAG **CTAAGGTAAC**CTTTATATATCTTGTGGAAAGGACGAAACA^1^
2.	Ion Torrent R.P. (tRP1 primer)	*CCTCTCTATGGGCAGTCGGTGAT* ^2^ATGAATACTGCCATTTGTCTCGACGTC^2^
3.	F.P. for gDNA PCR	GAGGGCCTATTTCCCATGATTC
4.	R.P. for gDNA PCR	GGTGGTGTGGTGTAAGG
5.	Oligo A for *SalI* adapter	TCGACCTCGAGACAAATGGCAGTATTC
6.	Oligo B for *SalI* adapter	GAATACTGCCATTTGTCTCGAGG

F.P.: Forward primer, R.P.: Reverse primer, *A sequence* ^*1*^, Key, **Barcode** (**one example**), Framework^1^, *P1 sequence* ^*2*^, Complementary to adapter^2^.

### Preparation of half-shRNA from the PCR product

Half of the hairpin present in shRNAs was removed by digesting the purified PCR product with *XhoI* (Fig. 3). To perform this digestion, 12 µg of PCR product was mixed with 120 µl of 10X FastDigest Buffer (Thermo Scientific), 60 µl of FastDigest *XhoI* (Thermo Scientific), and DNAase-free ddH_2_O to top up the volume to 1.8 ml reaction. The entire 1.8 ml was divided into 10 reactions with each reaction containing 180 µl. Reactions were incubated at 37 °C for 15 minutes on an Isotemp^TM^ Incubator (Thermo Scientific). The *XhoI* restriction enzyme reaction was performed immediately after PCR amplification to avoid cruciform formation. Digestion reactions were pooled together and purified with GeneJET PCR Purification Kit. PCR Purification Kit removes the smaller *XhoI*-digested product at this stage and larger *XhoI*-digested product was eluted in 20 µl DNAase-free ddH_2_O. Purified digested product was resolved (with 6X DNA Gel Loading Dye) at 90 V for 45 minutes in a Gel Electrophoresis Apparatus using 2% low-melting UltraPure™ Agarose (Thermo Scientific) gel stained with SYBR® Safe DNA Gel Stain in 1X TAE. The 316 bp expected band was detected on Gel Doc™ XR+ Imager using 100 bp DNA Ladder (some undigested product was also found at 359 bp). The 316 bp band was cut on a UV Transilluminator. The *XhoI*-digested product was purified by QIAquick Gel Extraction Kit according to manufacturer’s protocol and eluted in 20 µl DNAase-free ddH2O. DNA concentration and purity were quantified using a NanoDrop™.

### Preparation and ligation of the of *SalI* adapter to the *XhoI*-digested half-shRNA

To ligate the *SalI* adapter to the digested half-shRNA, we prepared a small double-stranded DNA using oligonucleotides 5 and 6 (Table 1). This adapter sequence has a common complementary sequence at 3′-end for hybridizing to the Ion Torrent RP primer (Fig. 3). In order to synthesize the adapter sequence, 10 µl of 200 µM Oligo A *SalI* adapter and 10 µl 200 µM Oligo B *SalI* adapter were added to 30 µl DNAase-free ddH2O to make the 50 µl annealing reaction. The reaction was heated at 98 °C, 80 °C, 70 °C, 60 °C, 55 °C, 50 °C, 40 °C, and 25 °C for 1 minute at each temperature using a Thermal Cycler and finally, 950 µL of DNAase-free ddH_2_O was added. DNA concentration and purity were quantified using NanoDrop™. *SalI* adapter from the previous step was ligated to *XhoI*-digested half-shRNA product (Fig. 3). The *SalI* adapter contains a cohesive compatible end (with *XhoI*-digested product) and common complementary sequence for hybridizing to the Ion Torrent RP primer at a later step. For the ligation reaction, 1:3 molar ratio of *XhoI*-digested half-shRNA product and *SalI* adapter was used (600 ng of *XhoI* digested PCR product was mixed with 5 µl of *SalI* adapter (42.7 ng/µl)) with 5 µl of T4 DNA Ligase (Invitrogen), 200 µl of 5X Ligase Reaction Buffer (Invitrogen) and DNAase-free ddH_2_O added to give a final volume of 1 ml, which was divided into 10 reactions. Reactions were incubated at 25 °C for 1 hour using an Isotemp^TM^ Incubator. Ligation reactions were pooled together and *SalI* adapter-ligated product was purified with the GeneJET PCR Purification Kit, which removes *SalI* self-ligated product as well. Ligated product was eluted in 25 µl DNAase-free ddH_2_O and DNA concentration and purity were quantified using a NanoDrop™.

### Barcode labeling of adapter-ligated half-shRNA product using Ion Torrent specific-primers

**Figure 4:**
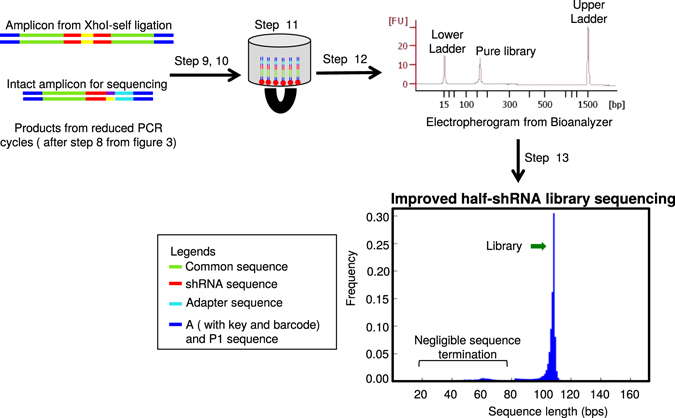
Reducing heteroduplex formation in half-shRNA library. Workflow showing steps to reduce heteroduplex formation in half-shRNA library. Step 1-8: same as in Fig. 3. Step 8: PCR purification of the barcoded PCR product. Step 9: *XhoI* digestion of the amplicon from *XhoI* self-ligated product. Step 10: PCR amplification after digestion of amplicon from *XhoI* self-ligation. Step 11: Magnetic bead-based purification of the barcoded product. Step 12: Quality assessment of the barcoded product on the Bioanalyzer. Step 13: Half-shRNA library sequencing on Ion Torrent platform. Representative agarose gel, the electropherogram from the Bioanalyzer, read-length histogram from Ion Torrent are shown. The sequence length is plotted in the X-axis and the frequency is plotted in the Y-axis. For half-shRNA, library is 108 bp excluding A and P1 sequence.

To multiplex sequence in the Ion Torrent sequencer, primers 1 and 2 (Table 1) with a barcoding sequence were attached to the ligated product (Fig. 3). To perform the barcode labeling, 30 µl of 10 µM Ion Torrent Barcode Forward Primer, 30 µl of 10 µM Ion Torrent reverse primer, 250 µl of 2X Phusion Master Mix with HF Buffer were assembled with 50 ng ligated product and DNAase-free ddH_2_O was added to give a final volume of 500 µl. The reaction was divided in 10 aliquots of 50 µl each and PCR was performed using a Thermal Cycler. The temperature profile for the PCR was 30 sec at 98 °C, 28 cycles of amplification (10 sec at 98 °C, 5 sec at 56 °C, 5 sec at 72 °C) and 15 sec at 72 °C. PCR reactions were pooled together and barcoded product was purified with the GeneJET PCR Purification Kit. Barcoded product was eluted in 25 µl DNAase-free ddH_2_O and DNA concentration and purity were quantified using a NanoDrop™. It is important to optimize the PCR reaction conditions at this step to reduce heteroduplex formation. PCR cycles were reduced from 28 cycles to 15 cycles to eliminate heteroduplex formation after optimizing the PCR cycles (Supplementary Fig. S1B). As 28 cycles (as in Fig. 3; represented by bioanalyzer data) but not 15 cycles (as in Fig. 4) formed heteroduplex, PCR reactions were performed with 15 cycles.

### Removal of *XhoI* and self-ligated product

To eliminate the *XhoI* self-ligated product, *XhoI* digestion was performed. Briefly, 1 µg of barcoded product was mixed with 10 µl of 10X FastDigest Buffer and 5 µl of FastDigest *XhoI* and the final volume was topped up to 150 µl using DNAase-free ddH_2_O. Digestion reaction was incubated at 37 °C for 15 minutes using an Isotemp^TM^ Incubator. After the removal of the self-ligated products, the barcoded PCR product was purified with the GeneJET PCR Purification Kit and DNA was eluted in 50 µl of DNAase-free ddH_2_O. For the lower cycle barcoded product (15 cycles), magnetic bead-based purification was done instead of gel extraction to reduce sample loss (Fig. 4). Briefly, the eluted product was purified with Agencourt® AMPure® XP Reagent (Beckman Coulter) for magnetic bead-based purification according to manufacturer’s protocol. This was a two-step purification method where the first step removed all the high molecular weight DNA contamination (e.g. genomic DNA) and the second step removed all the smaller DNA fragments (e.g. primer, primer dimer, and restriction digested fragments). In both cases, DNA concentration and purity were quantified using NanoDrop™.

### Quality assessment of library from different methods

Throughout all procedures described above, the quality of the library sample or any PCR product was assessed with the Agilent 2100 Bioanalyzer, using the Agilent High Sensitivity DNA chip, according to the manufacturer’s protocol. For this purpose, 1 µl of 500 pg/µl sample was applied to the chip. A sharp peak was expected for a pure library sample. Presence of multiple peaks and/or broad peak larger than the expected library suggested formation of a heteroduplex.

### Ion Torrent sequencing, data processing and analysis

Amplicon concentration was determined using a NanoDrop™ and 25 µl of DNA (26 pM) was prepared for emulsion PCR. Emulsion PCR was performed using the Ion PGM™ Hi-Q™ OT2 Kit (Life Technologies), according to manufacturer’s protocol. First, a unique DNA amplicon was amplified and bound to a single Ion Sphere Particle (ISP) by emulsion PCR. Amplification primers that bind to A and P1 adapters were used for clonal amplification so that each ISP was covered with many copies of the same DNA fragment. Second, because the A primer was biotinylated, template positive ISPs could be isolated using Ion Torrent enrichment beads and non-templated ISPs were removed. Third, dsDNA anchored to the ISPs was denatured. This allowed the ISPs with ssDNA to go into solution while the biotinylated strand remain bound to enrichment beads. The solution containing ssDNA enriched ISPs was used for next generation sequencing.

Next generation sequencing was performed using the Ion PGM™ System (Thermo Scientific). Ion 318 chips and Ion PGM Hi-Q Sequencing Kits were used according to manufacturer’s protocol. Base calling, chip analysis, and barcode separation were performed using the Ion Torrent Server software version 5.0. Chip analysis included percentage ISP loaded, percentage of enriched ISPs, percentage polyclonal reads (ISPs with multi-type DNA templates), and percentage of low quality reads. Total raw sequences were retrieved as FASTQ format from Ion Torrent server and counted and plotted afterwards.

Queries on each Ion Torrent read was done with a computational tool for pattern recognition called regular expression (regex)^39^. Each read was scanned for an established known set of sequence (represented as framework in Table 1) followed by the number of nucleotides equal to the length of the library sequences (in our case, 21) and then the cleaved-*XhoI* site (‘CTC’). Once all reads had been scanned, counts of each unique sequence were saved to a file using script (python-2.7.9) provided as additional file named as “Script file” according to the method described in Supplementary Fig. S4A.

### Sequencing library preparation for GeCKO library

**Figure 5:**
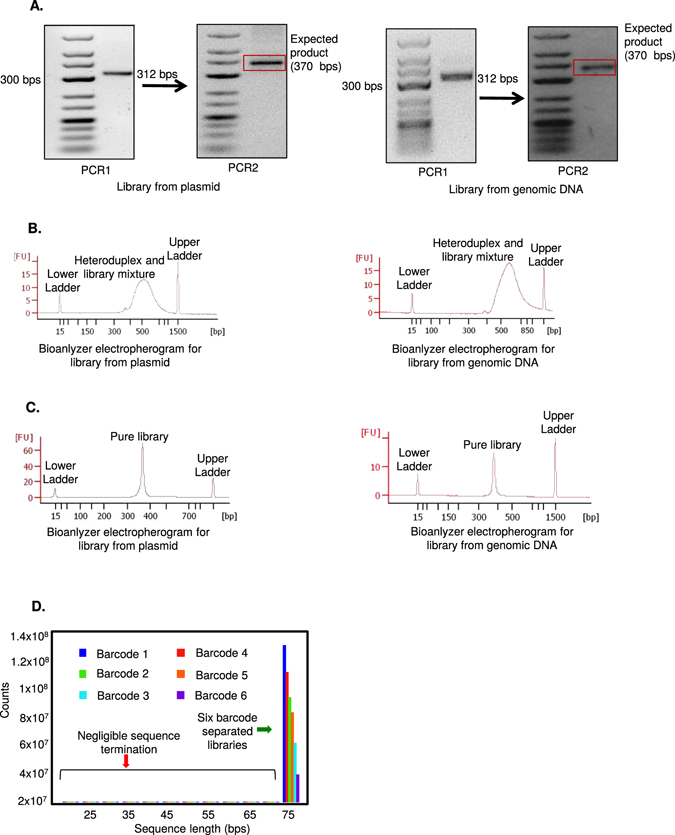
Steps involved in sequencing GeCKO library. (**A**) Gel image of barcoded library preparation of Gecko library from plasmid or gDNA. (**B**) Bioanalyzer electropherogram analysis showing formation of heteroduplex in barcoded CRISPR library from both plasmid and gDNA. (**C**) Bioanalyzer electropherogram analysis showing elimination of heteroduplex formation in CRISPR library from both plasmid and gDNA by reduced PCR cycles. (**D**) Read-length histogram showing Illumina sequencing results for the GeCKO library amplified from plasmid. CRISPR library is 76 bp after barcode separation. The sequence length is plotted in the X-axis and the sequence count is plotted in the Y-axis. Six different barcodes represent six different dilutions of the library, where consecutive dilutions cause comparatively lower library counts.

For quality assessment of the GeCKO library from plasmids^19^, PCR was performed as described by Shalem *et al*. Briefly, two steps of PCR reactions were performed. For the first PCR (PCR 1) using naive plasmid DNA library, 20 µl of 10X Pfx Amplification Buffer, 20 µl of 10X PCR_X_ Enhancer Solution, 3 µl of 10 mM dNTPs, 4.5 µl of 20 uM Primer Mix (using primer 1 and 2 from Table 2), 3 µl of 50 mM MgSO_4_ and 1.5 µl of Platinum™ Pfx DNA polymerase were added together with 40 ng of GeCKO library. DNAase-free ddH_2_O was added to give a final volume of 100 µl. The reaction was divided in 50 µl aliquots and PCR was done using a Thermal Cycler. The temperature profile for the PCR was 5 min at 98 °C, 30 cycles (Note that this cycle number was reduced as described below) of amplification (15 sec at 98 °C, 15 sec at 65 °C, 40 sec at 72 °C), and 5 min at 72 °C. The same reaction was done with multiple dilutions of the same library using different barcodes. PCR reactions were pooled together and resolved (with 6X DNA Gel Loading Dye) at 100 V for 1 hour in a Gel Electrophoresis Apparatus using 2% low-melting UltraPure™ Agarose gel stained with SYBR® Safe DNA Gel Stain in 1X TAE. The expected 312 bp band was visualized using 50 bp DNA Ladder on a UV Transilluminator (Fig. 5A). For the second PCR (PCR 2) 5 µl of PCR 1 was used as a template. Briefly, 20 µl of 10x Pfx Amplification Buffer, 20 µl of 10x PCRx Enhancer Solution, 3 µl of 10 mM dNTPs, 4.5 µl 20 µM Primer mix (using primer 3 and 4 from Table 2), 2 µl of 50 mM MgSO_4_, and 1.5 µl of Platinum™ Pfx DNA Polymerase were added together with 10 µl of amplicon from PCR1 (for a 2X reaction) and DNAase-free ddH_2_O was added to give a final volume of 100 µl. The reaction was divided in 50 µl aliquots and PCR was performed using a Thermal Cycler. The temperature profile for the PCR was 5 min at 94 °C, 25 cycles (Note that this cycle number was reduced as described below) of amplification (15 sec at 94 °C, 30 sec at 63 °C, 23 sec at 72 °C), and 5 min at 72 °C. PCR annealing temperature and cycle number optimization for Illumina primers was done (Supplementary Fig. S1C,D). PCR reactions were pooled together and resolved (with 6X DNA Gel Loading Dye) at 100 V for 1 hour in a Gel Electrophoresis Apparatus using 2% low-melting UltraPure™ Agarose gel stained with SYBR® Safe DNA Gel Stain in 1X TAE. The expected 370 bp band was excised, using 100 bp DNA Ladder as a guide, on a UV Transilluminator (Fig. 5A). QIAquick Gel Extraction Kit was used to purify the library. DNA was eluted in 30 µl DNAase-free ddH_2_O and DNA concentration and purity were quantified using a NanoDrop™. The quality of samples was assessed on Bioanalyzer as previously described for shRNA library (Fig. 5B). Since Bioanalyzer data showed formation of heteroduplex structures, we reduced the PCR cycles. Specifically, we found that reducing first PCR cycle number to 18 cycles and the second PCR cycle number to 20 cycles eliminated heteroduplex formation (Fig. 5C). We sequenced the GeCKO library using Illumina NextSeq 500 High Output (75 Cycles, 400 M Reads). For assessment of the GeCKO library from genomic DNA, the PCR was performed as described above. However the genomic DNA template concentration was set at 30 µg to achieve higher representation of the integrated library.

**Table 2:** Oligonucleotides for CRISPR library preparation (Integrated DNA Tech.).

ID	Oligonucleotide (5′-3′)	Sequences
1.	F.P. for gDNA PCR	AATGGACTATCATATGCTTACCGTAACTTGAAAGTATTTCG
2.	R.P. for gDNA PCR	CTTTAGTTTGTATGTCTGTTGCTATTATGTCTACTATTCTTTCC
3.	Illumina F.P.	*AATGATACGGCGACCACCGAGATCTACACTCTTTCCCTACACGACGCTCTTCCGATCT* T **AAGTAGAG**TCTTGTGGAAAGGACGAAACACCG
4.	Illumina R.P	*CAAGCAGAAGACGGCATACGAGAT* **AAGTAGAG** *GTGACTGGAGTTCAGACGTGTGCTCTTCCGATCT* TTCTACTATTCTTTCCCCTGCACTGT

F.P.: Forward primer, R.P.: Reverse primer, *Illumina adapter*, Stagger, **Barcode** (**one example**), Priming site.

### Availability of data and materials

The datasets used and/or analyzed during the current study available from the corresponding author on reasonable request.

## Results

### Efficient sequencing of shRNA libraries was hindered by secondary structures

Sequencing of shRNA, sgRNA, and other mixed-oligo based pooled libraries is a challenging task due to sequencing failure caused by the formation of secondary structures and/or mixed heteroduplex templates (Fig. 1). To improve sequencing throughput, we used a small shRNA library of about 15,000 shRNAs and performed a pooled screen as previously described^5, 6, 38^. Lentivirus particles that express shRNAs were used to transduce target cells and genomic DNA was isolated. Approximately 3 million transduced cells were used in the screening process to maintain ~200 fold representation for the shRNA library. We amplified these shRNA sequences from the genomic DNA. While the PCR product may show a single band on an agarose gel, often times they are enriched with heteroduplex formation as well as cruciform formation. During sequencing reaction, these secondary structures often result in incomplete, low quality, and polyclonal reads (Fig. 1). We used an Ion PGM^TM^ system to perform our sequencing reactions. In principle, after clonal amplification, multiple copies of a single template type from the shRNA library should surround each Ion Sphere Particle (ISP). During sequencing, all copies of the template on ISPs should be single-stranded (in each well of the sequencing chip) and anneal to the sequencing primer (included in the Kit). During this annealing, single-stranded template can form a hairpin structure on some ISPs, which results in termination of the polymerization required for sequencing. This hairpin structure causes de-synchronization of the incorporation of a new nucleotide in the synthesizing strands between clonal fragments attached to a single ISP. In the worst case, inappropriate nucleotide insertion at one or several flows results in phasing of a segment of the cluster, which will lead to either polyclonal or low quality reads. On the other hand, heteroduplex formation results in ISPs being surrounded by two different templates, which increases the rate of polyclonality and generates many polyclonal and low quality reads. As a result, incomplete, low quality, and polyclonal reads are found in the final output (Fig. 2). As clearly evident from the read length histogram of our shRNA library, only 21.2% of the sequence output was usable reads. Of this, only 83.96% of our 15 K shRNA library was covered. Disappointingly, this coverage represented only 10-fold of our library and is not sufficient for extensive analysis. Interestingly, analysis of the incomplete reads revealed that termination of these sequences starts at around 30 bp, which corresponds to the beginning of the hairpin loop (Fig. 2). This indicated that formation of hairpin structures was responsible for this premature termination. A second major peak of terminated sequences also occurred around 65 base pair (Fig. 2). As each variable region begins at 57 bp, it would appear that terminations at this point result from the Ion Torrent software dropping reads due to the low quality score associated with misidentified polyclonal ISPs. Overall, our initial analyses strongly indicated that efficient sequencing of shRNA libraries was hindered by secondary structures on Ion PGM^TM^ system.

### Sequencing of adapter-ligated, half-shRNA improved the quality of results

Sequencing of shRNA library results in abundant sequence termination. This becomes a major issue when this procedure is carried out in medium-throughput sequencers such as the, Ion PGM™ System. In an effort to reduce this effect, we cleaved the shRNA sequence in half by taking advantage of the *XhoI* restriction site present within these sequences^40^. In order to amplify these half-shRNA sequences, we designed an additional *SalI* adapter and ligated this with the half-shRNA to generate an adapter-ligated half-shRNA product (Fig. 3). During the ligation of *SalI* adapter to *XhoI-*digested product, self-ligated products were formed between *XhoI*-digested products or *SalI* adapters (Fig. 3). The *SalI* self-ligated product was removed during the PCR purification step. However, to eliminate the *XhoI* self-ligated product, *XhoI* digestion was performed on the ligated product, followed by PCR purification and gel extraction. *XhoI* (C/TCGAG) and *SalI* (G/TCGAC) recognition sites are compatible to each other and when ligated they create a unique site (CTCGAC), which cannot be recognized by *XhoI* and *SalI* enzymes. This new site makes it possible to remove only the *XhoI* self-ligated product but not the expected product. Unfortunately, the adapter-ligated half-shRNA product did not generate a sharp clean peak when analyzed using the Bioanlyzer (Fig. 3). We assumed that this artifact observed in the Bioanalyzer analysis could be generated by heteroduplex formation. Consistent with the Bioanalyzer data, we found that the hairpin effect was completely eliminated as shown by the loss of peak at 30 bp read length histogram (Fig. 3) where the half-shRNA sequences increased the count of sequences of the expected library (45.9% of the sequence output was usable read compared to 21.8% in full hairpin sequencing). We also found that these sequences covered 94.7% of our 15 K shRNA library sequences with a 26-fold representation. However, the second major peak of terminated sequences around 65 base pairs, still remained suggesting the existence of heteroduplex structures (Fig. 3). Therefore, we next set out to eliminate those failed sequences derived from heteroduplex formation.

To address the heteroduplex formation, we reduced PCR cycles as has been previously suggested^26–30^ from 28 to 15 cycles. As the diversity greatly varies among libraries, it should be noted that, this step requires to be optimized based on the nature of the library used. The reduction in PCR cycles decreased the yield of the DNA and so we used a magnetic bead-based purification (Agencourt AMPure XP) to minimize DNA loss (Fig. 4). This solid phase reverse immobilization technique is advantageous for low concentration DNA clean up and we successfully obtained a 95% yield after purification. This modification eliminated the heteroduplex formation and resulted in a sharp clean preparation of the library, as seen in the Bioanalyzer analysis (Fig. 4). Consistent with this, we found the peak at 65 bp was reduced and the read length histogram (Fig. 4) showed that the PCR optimization removed the formation of heteroduplex substantially and increased the expected library reads (82.5% of the sequence output compared to 45.9% of just half-hairpin sequencing or 21.8% in full-hairpin sequencing) (Fig. 4). These sequences covered 98.7% of our 15 K shRNA library with a 55-fold representation. Overall, our results suggest that we reduced a considerable amount of non-usable reads after the elimination of hairpin and heteroduplex formation (Figs 2–4; Supplementary Fig. S2A–C).

To show that our optimal conditions eliminate heteroduplex structures, we also used a pooled GeCKO (Genome-scale CRISPR Knock Out) library^19^ that has recently become a powerful tool to query the genome for complete loss of function as opposed to shRNA libraries that function in hypomorphic context^4^. As these CRISPR libraries are originally designed from mixed-oligo PCRs, they also suffer from the formation of heteroduplexes structures (Fig. 5A–C). Although these are much larger libraries and might not be compatible with Ion PGM^TM^ sequencing due to limited throughput, we expected that elimination of heteroduplex structures might at least improve the multiplexing capabilities in medium throughput instruments such as Illumina Next Seq 500. Therefore, we extended our method as a validation strategy by using the GeCKO library prior to Illumina sequencing. In addition, a detailed and validated protocol in preparing CRISPR libraries for Illumina sequencing will also benefit researchers as such resources are still limited. Sequencing of the GeCKO library requires two steps of PCR reactions. Reduction of the cycles for both PCR reactions to amplify the library from plasmid or genomic DNA, decreased the ~520 bp DNA smear and resulted in an increase of the ~370 bp product (Fig. 5B,C; Supplementary Fig. S3). Sequencing of the GeCKO library from plasmid on the Illumina platform by adapting our procedure showed minimal amounts of incomplete reads, indicating that our method was equally applicable for larger libraries as well (Fig. 5D).

**Figure 6:**
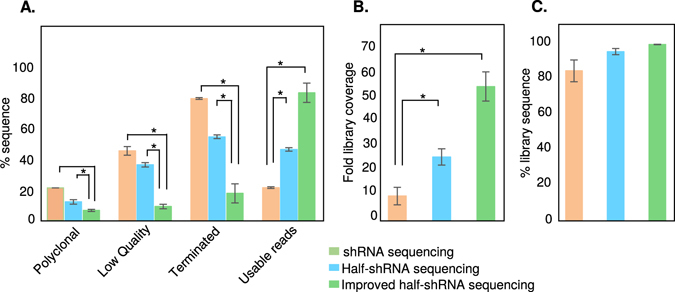
Comparison of the quality of sequencing across different methods. (**A**) Comparison of metrics from shRNA, half-shRNA and improved half-shRNA library sequencing. Polyclonal reads are presented as percentage of initial loading and enrichment. Low quality reads are presented as percentage of total sequence output after loading, enrichment and initial polyclonal removal. Terminated and barcoded library reads are presented as percentage of total filtered reads by Ion Torrent server. (**B**) Comparison of library fold coverage from shRNA, half-shRNA and improved half-shRNA library sequencing. (**C**) Comparison of identified library sequences from shRNA, half-shRNA and improved half-shRNA library sequencing. *p < 0.05.

Overall, across the three methods (Figs 2–4; Supplementary Fig. S2A–C), it was clearly observed that polyclonal, low quality, and terminated sequences were gradually decreased, while the intended library reads were increased (Fig. 6A). The library-fold coverage was also increased though there was no comprehensive difference among identified library sequences by these methods (Fig. 6B,C). Importantly, our method did not affect the library distribution, as the normalized library reads remained the same irrespective of template origin or sequencing procedures (Supplementary Fig. S4B). Thus, the evenness (calculated as standard deviation of library counts/mean library count) was not influenced by the modification of the method (Supplementary Table S1). In addition, we also provide script file to identify library sequences based on the method described in Supplementary Fig. S4A.

## Discussion

Direct shRNA library sequencing from genomic DNA generally causes polyclonal, low quality, and incomplete reads due to hairpin formation during sequencing. This challenge is significantly amplified in low-to-medium throughput bench-top sequencers as their coverage is compromised. As shRNA libraries are designed to form functional hairpin structures that when processed within the cell, knockdown the expression of the intended gene, it is natural that these sequences form hairpin structures during sequencing reactions and reduce sequence quality, irrespective of Ion Torrent or Illumina platforms. Similarly, heteroduplex structures can also reduce sequence quality. This heteroduplex issue is not unique to shRNA libraries alone but affects all types of mixed-oligo libraries (e.g. CRISPR, phage display libraries, etc.). For example, Rebollo *et al*., showed that 70% of sequences could not be read due to heteroduplex in a phage-selected peptide library^29^. Similarly, 40% of reads from Illumina sequencing of genetically distinct HIV-1 genome were unexpected recombinant sequences due to heteroduplex formation^31^. These secondary structures also pose an important challenge when sequencing larger libraries from a multiplexing point of view. Unfortunately, computational predictions to simulate the formation of heteroduplex structures are also limited due to high randomness associated with the formation of these structures.

Our method aims to eliminate sequence failure and maximize throughput in low-to-medium throughput bench-top sequencers. We show that the adapter-ligated half-shRNA sequencing increases usable read output by ~60% relative to current sequencing strategies (Fig. 6A). To confirm that our experimental procedures successfully eliminated the hairpin related issues, we carried out sequencing of both full-shRNA and half-shRNA library samples. We showed that sequencing half-shRNA reduced the polyclonal, low quality, and sequence termination while increasing reads of the intended library (Figs 2 and 3). In addition, reduction of heteroduplex structures also maximized our throughput (Fig. 4). As increased-fold representation of the shRNA library is important for the reproducibility of pooled shRNA screens^41^, our methodology should alleviate any of these concerns because sequencing of half-hairpins increased the fold coverage by at least five times (Fig. 6B).

Our method resulted in improvements in the initial quality assessment by the server software (after loading and enrichment). While shRNA sequencing showed 21% initial polyclonal reads and 45% low quality reads, half-shRNA sequencing reduced them to 12% and 36%, respectively (Fig. 6A). After heteroduplex removal, half-shRNA sequencing decreased the polyclonal and low quality reads further to 6.5% and 9%, respectively (Fig. 6A). In comparison to some of the established methods such as MuPlus (transposon-based), MuSeek (commercial), and other ligation-based methods, our approach showed a significant reduction in the rate of polyclonal reads. While these methods exhibited 23%, 51%, and 31% polyclonal reads, respectively, our method produced only 6.5%^26^. Our method also reduced low quality reads (9%) in comparison to the commercial MuSeek method (55%), though MuPlus and other ligation-based methods have similar levels of efficiency (MuPlus: 6% and other ligation-based: 9%)^26^. Additionally, final sequence output after loading, enrichment, initial polyclonal removal, and low quality removal have been significantly increased with our method (88%), when compared to other methods (MuPlus: 72%, MuSeek: 22% and other ligation-based method: 63%)^26^. Unlike previously used sequencing strategies^42^, our ligation-based, half-shRNA sequencing is readily amenable to any pooled shRNA screening studies. Our strategy to detect and minimize heteroduplex formation in shRNA or CRISPR libraries can also be extended to any mixed-oligo libraries.

As targeted screening of smaller libraries such as those of the kinome etc., using low-to-medium throughput bench-top next generation sequencers demands minimal unusable reads that arises from hairpin and/or heteroduplex formation, we expect that our approach will be beneficial. That being said, our method can also be extended to whole genome screening using IIlumina platforms. With the recent increase in the library sizes like the ultracomplex-pooled shRNA libraries with 25 shRNAs per gene^1^ or the TKO library, where 10 guide RNAs target each gene^3^, there is an ever-increasing demand on the sequencing throughput. Therefore, in its current state, maximizing the sequencing strategy is becoming a pressing issue. A whole genome screen using a 90 K shRNA pool may require ~45 million reads for ~500-fold coverage for a single time point. In fact, a single screen requires multiple time points and replicates, easily exceeding the limitations of a bench-top sequencer (e.g. Ion PGM^TM^). While Illumina sequencing technologies provides vast coverage, over 40% to 50% of the reads are non-usable, we expect that our methodological improvements will minimize the loss of reads and maximize throughput and multiplexing capabilities.

## Electronic supplementary material

Supplementary information

Supplementary Dataset
