# Supplementary material for: Enhancing the throughput and multiplexing capabilities of next generation sequencing for efficient implementation of pooled shRNA and CRISPR screens
Source: Sci Rep. 2017 Apr 21;7:1040. doi: 10.1038/s41598-017-01170-z (PMC5430825; doi:10.1038/s41598-017-01170-z)
Supplement: Supplementary file 1 — Supplementary information [file 41598_2017_1170_MOESM1_ESM.pdf]

Enhancing the throughput and multiplexing capabilities of next generation sequencing for efficient implementation of pooled shRNA and CRISPR screens

Md. Fahmid Islam[1], Atsushi Watanabe[2,3], Lai Wong[1], Conor Lazarou[2], Frederick S. Vizeacoumar[2], Omar Abuhussein[4], Wayne Hill[2], Maruti Uppalapati[2], C. Ronald Geyer[2*], Franco J. Vizeacoumar[2,4,5*]

[1]Department of Biochemistry, University of Saskatchewan, Saskatoon, S7N 5E5, Canada

[2]Department of Pathology, University of Saskatchewan, Saskatoon, S7N 0W8, Canada

[3]Department of Hematology, Nephrology and Rheumatology, Graduate School of Medicine, Akita University, Akita, Japan

[4]College of Pharmacy and Nutrition, University of Saskatchewan, Saskatoon, S7N 5C9, Canada

[5]Cancer Research, Saskatchewan Cancer Agency, 107 Wiggins Road, Saskatoon, S7N 5E5, Canada

*Corresponding authors

Franco J. Vizeacoumar. Tel: 306-966-7010; email: franco.vizeacoumar@usask.ca

C. Ronald Geyer. Tel: 306-966-2040; email: ron.geyer@usask.ca

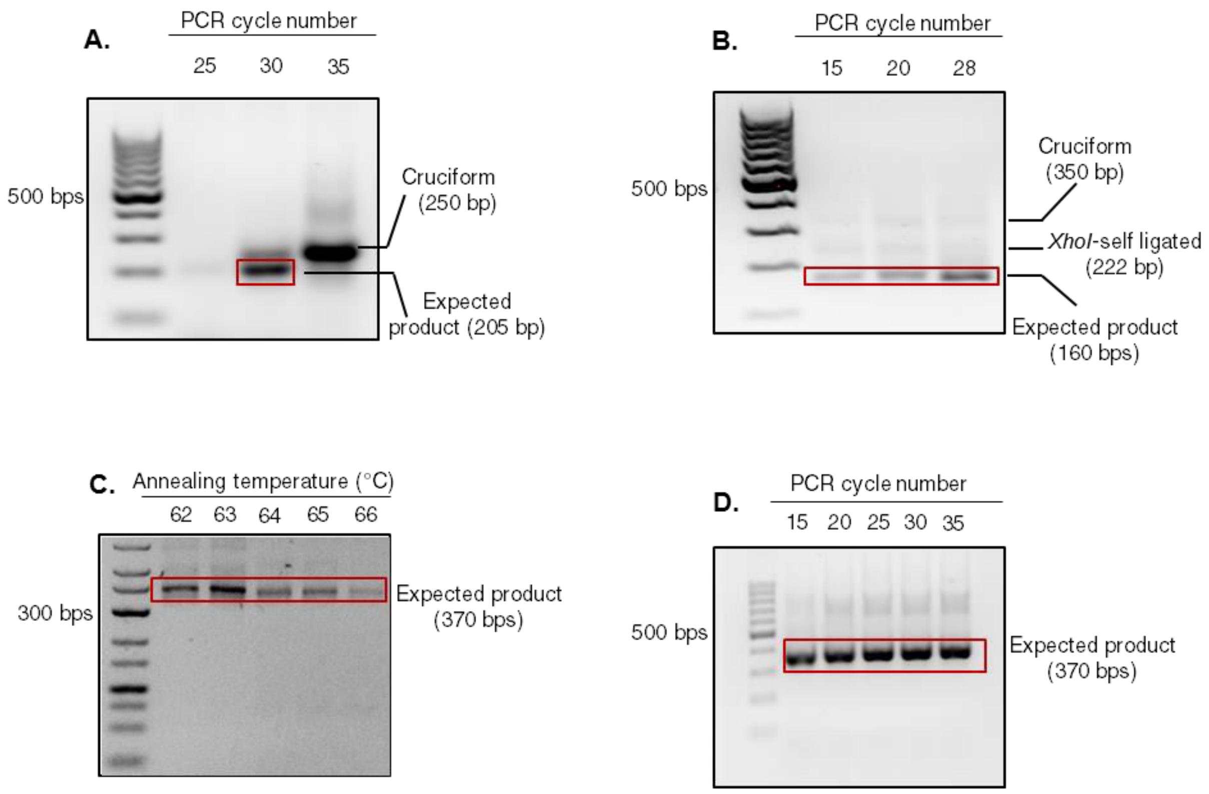

Supplementary Figure S1: PCR optimization of shRNA library and GeCKO library preparations

(A) PCR cycle number optimization for shRNA library amplification from gDNA (100 bp ladder). **(B)** PCR cycle number optimization for reducing heteroduplex formation during amplification of half-shRNA library (100 bp ladder). **(C)** Annealing temperature optimization for GeCKO library amplification from plasmid using Illumina primers (50 bp ladder). (D) Cycle number optimization for GeCKO library amplification from plasmids (100 bp ladder).

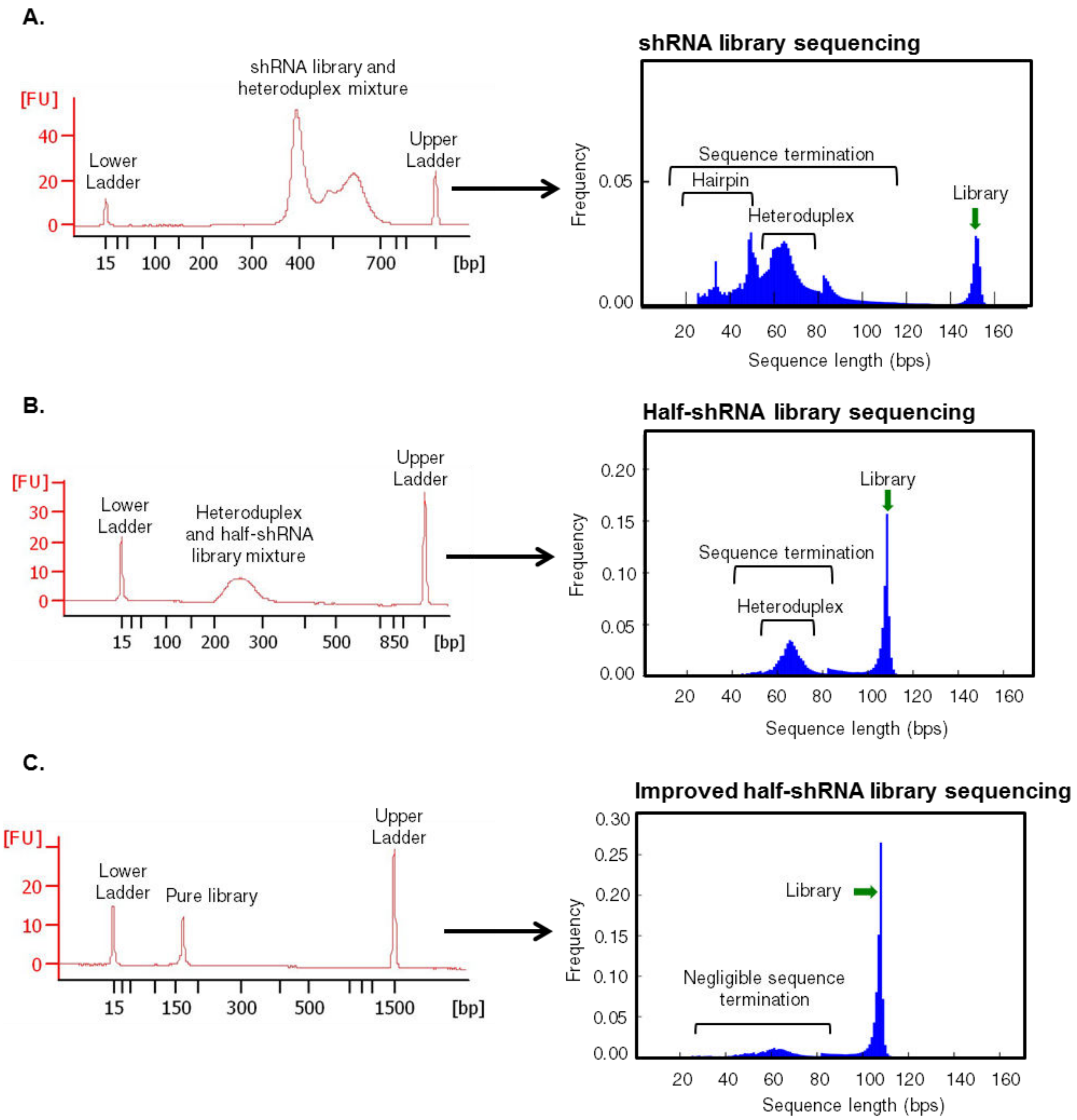

Supplementary Figure S2: Results from replicate experiments for shRNA library preparation in different methods

(A) Bioanalyzer data and read length histogram for shRNA library sequencing from gDNA. **(B)** Bioanalyzer data and read length histogram for half-shRNA library sequencing. **(C)** Bioanalyzer data and read length histogram for half-shRNA library sequencing with improved method.

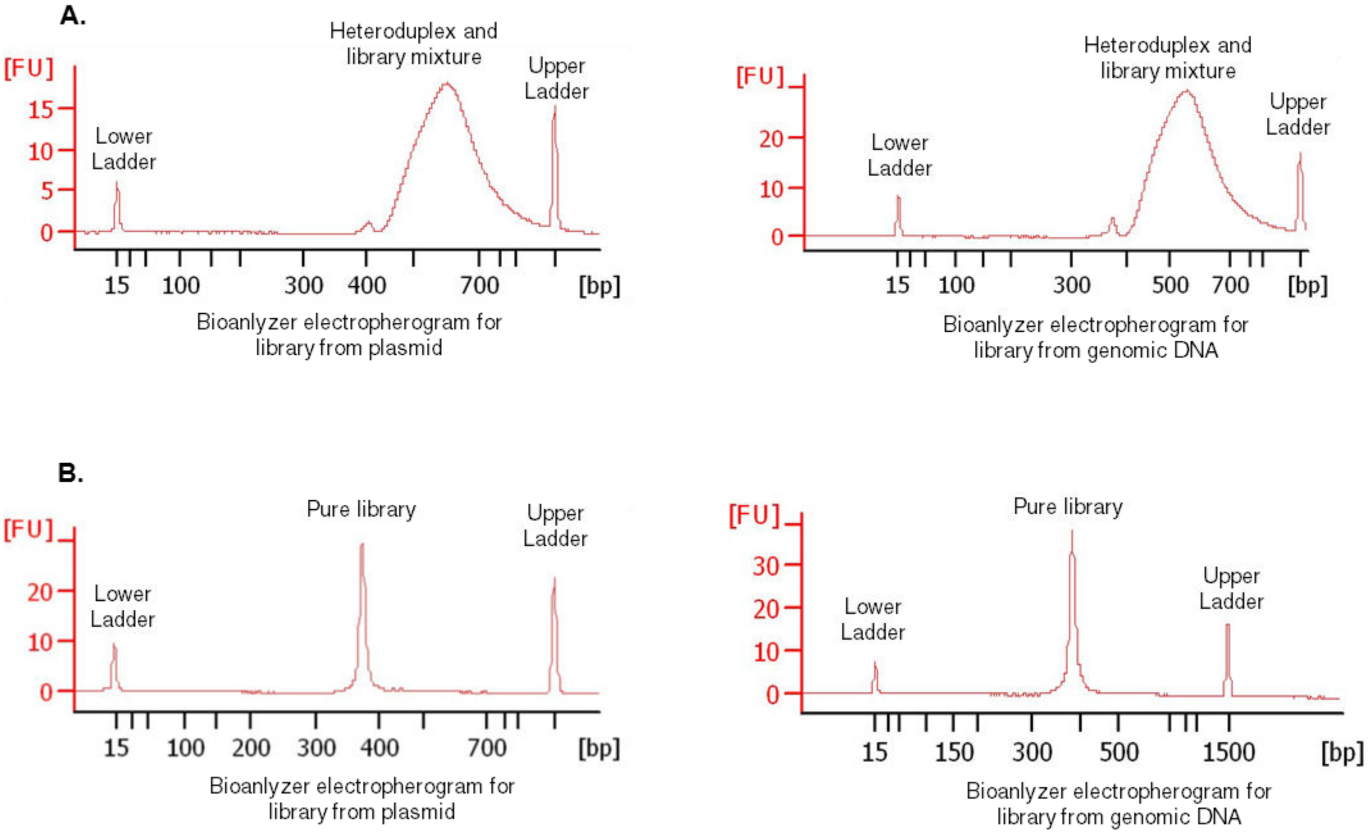

Supplementary Figure S3: Results from replicate experiments for GeCKO library preparation

(A) Bioanalyzer data indicating heteroduplex formation in GeCKO library preparation both from plasmid and gDNA. **(B)** Bioanalyzer data indicating heteroduplex removal in GeCKO library preparation both from plasmid and gDNA after the optimization.

A.

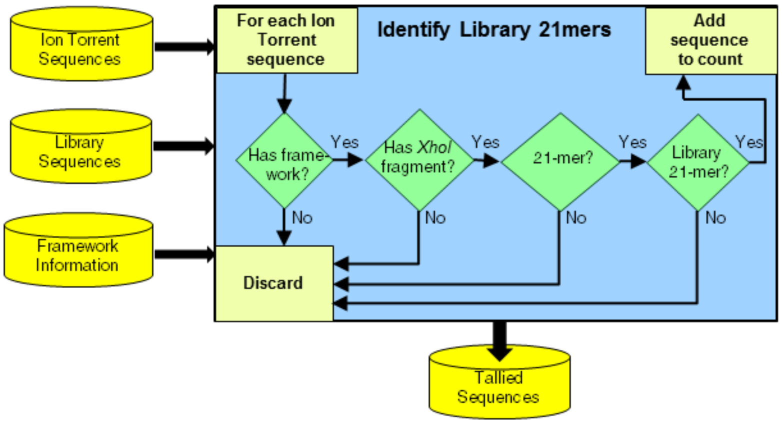

B.

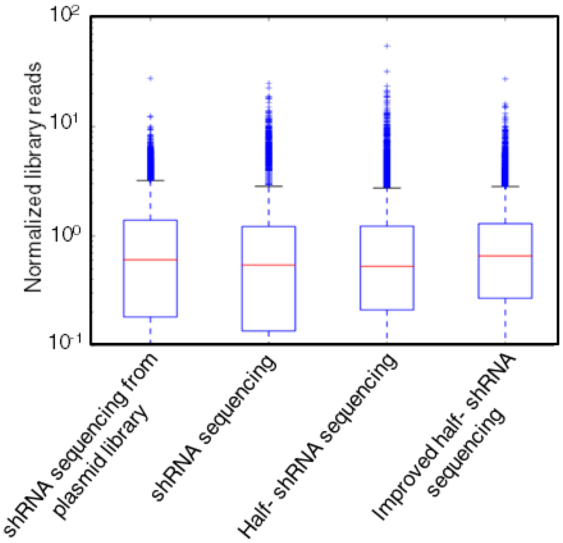

Supplementary Figure S4: Data analysis outline and library distribution analysis

(A) A flowchart to analyze results from half-shRNA library sequencing. **(B)** Comparison of library distribution found from shRNA library sequencing from plasmid library, and shRNA, half-shRNA and improved half-shRNA library sequencing from genomic DNA of initial time point of screening (based on $\log_{10}$ normalized value of the library sequence count).

Supplementary Table S1: Comparison of library evenness across different methods based on plasmid library and initial time point of screening

Library type	Standard deviation of library sequence counts/mean library sequence count
shRNA sequencing from plasmid library	1.2
shRNA sequencing	1.5
Half-shRNA sequencing	1.5
Improved half-shRNA sequencing	1.2
